# Acute esophageal necrosis: a case report and review

**DOI:** 10.11604/pamj.2013.14.109.2000

**Published:** 2013-03-19

**Authors:** Mounia Lahbabi, Adil Ibrahimi, Nouredine Aqodad

**Affiliations:** 1 Department of Hepato Gastroenterology Hassan II University Hospital Fes, Morrocco

**Keywords:** Black oesophagus, acute oesophageal necrosis, necrotizing esophagitis, gastrointestinal haemorrhage, ischemia

## Abstract

Acute esophageal necrosis, commonly referred to as “black esophagus” or “acute necrotizing esophagitis”, is a rare clinical disorder with an unclear etiology. The definition excludes patients with a history of recent caustic ingestion. Oesophageal necrosis can be diagnosed at endoscopy by the presence of black necroting appearing oesophagus. Contrary to the caustic oesophagitis whose treatment is often surgical, treatment of the acute necrositing oesophagitis is primarily medical. The prognosis for patients who develop acute necrotizing oesophagitis is generally poor. We report a new case of acute necrotizing oesophagitis and undertook a literature review of this rare diagnosis.

## Introduction

Esophagitis can be complicated in its most severe cases, by a total necrosis of the esophageal mucosa, leading to “black esophagus” as described by endoscopists [1]. Acute necrotizing esophagitis is rare clinical entity with an unclear etiology, its pathogenesis remains unknown, and most investigators have suggested an ischemic origin based on histopathologic and clinical data [2]. We report a new case of black esophagus in patient admitted for hematemesis in a state of septic shock, and we will be discussing the literature surrounding this rare entity.

## Patient and observation

**Figure 1:**
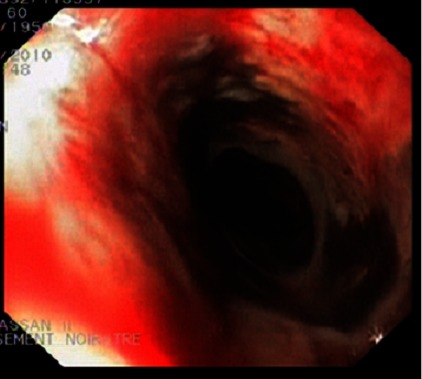
Endoscopic view showing of the upper third of the esophagus, which is circumferentially congestive associated with diffuse bleeding

**Figure 2:**
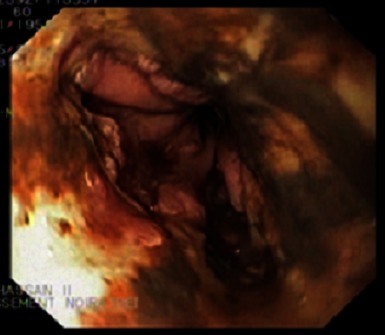
Endoscopic view showing of the middle and lower third of the esophagus, which had circumferentially black pigmentation: the mucosa was black and covered by an exudate of the same color

A 60 year-old man presented to an outside institution for septic shock with hematesis. He had a medical history of diabetes mellitus, hypertension and he was amputated right leg (trans-femoral amputation) for diabetic arteriopathy six months before admission complicated by venous thrombosis. Home medications included daily pioglitazone, atenolol, furosemide and anticoagulant with poor compliance. Initial examination revealed a patient in state of septic shock, respiratory rate 28 cycles per min, his pulse was regular with an apical rate of 120 beats/min, temperature 39° C, blood pressure 70/40 mmHg, he had necrotic and suppurative amputation stump with peripheral pulse abolished. Patient was given immediately oxygen, fluids, antibiotics, and drugs to increase blood pressure. Six hours later, the patient presented a single episode of hematemesis. There was no associated melena or abdominal pain. He had no history of alcohol use, liver disease, varices, peptic ulcer disease, abdominal aortic surgery, nonsteroidal anti inflammatory drug use, gastroparesis, or previous GI bleeding. Physical examination was unremarkable. Pertinent laboratory studies included a hemoglobin level of 10 g/dL, platelet count was normal, blood urea of 1,2 g/l (0,18-0,45 g/L), and a creatinine level of 68 mg/L (7-13 mg/L). After hemodynamic stabilization, an oesophageo-gastro-duodenoscopy was performed which showed: The upper third of the esophagus was circumferentially congestive (Figure 1), but the middle and lower third showed circumferential black pigmentation: the mucosa was black and covered by an exudate of the same color associated with diffuse bleeding (Figure 2). Gastric mucosa was strictly normal in direct vision and in retrovision, the bulb and duodenum were normal. Biopsie specimens were showed necrotic debris, mucosal submucosal necrosis with a local inflammatory response. The treatment of this condition was based continuous high dose omeprazole (8 mg / h) after bolus of 80mg and total parenteral nutrition. The patient experienced no further hematemesis or melena. Due to the severity of the necrosis, and with deterioration of his condition and persistent sepsis he died later in the same day.

## Discussion

Black esophagus, also known as acute necrotizing esophagitis, was first described by Goldenberg et al in 1990 [3]. Ben Soussan described an incidence of 0.2% (8 cases from 3900 endoscopies) [4], and while Grudell reported an incidence of 0.008% (6 cases in 78 847 endoscopies)[5]. Gurvits reviewed the literature until 2006, finding a total of 88 cases [6]. Acute esophageal necrosis clearly shows sex and age predilection. Men are four times more commonly affected than women, and although the disease has been documented in every age group, the peak incidence occurs in the sixth decade of life with an average age of 67 years [7]. Endoscopically, the lesion often appears circumferential and black, with friable or macerated mucosa usually involving the distal two thirds of the esophagus that stops abruptly at the gastroesophageal junction [6].

The diagnosis can be based on the typical endoscopic appearance after excluding ingestion of corrosive agents based on history and absence of evidence of oropharyngeal injury [8]. Histopathology usually shows necrotic debris, mucosal submucosal necrosis with a local inflammatory response [7]. As in our patient, the most common clinical presentation of black esophagus is hematemesis and melena [6]. The etiology of black esophagus is unknown. The condition is thought to be multifactorial. Conditions associated with black esophagus are advanced age, male sex, diabetes, hemodynamic compromise, hypercoagulable state, trauma, gastric volvulus, myocardial ischemia, some viral and fungal disease (*Klebsiella pneumonia*, cytomegalovirus, herpes simplex virus, *Penicillium chrysogenum*, Candida), alcoholic hepatitis, acute renal failure, severe acid reflux, hypoxemia, malnutrition, Stevens-Johnson syndrome, hematoma from traumatic transection of thoracic aorta, polyarteritis nodosa, pulmonary lobectomy with paraesophageal lymph node dissection, severe infectious mediastinitis, emphysematous gastritis and acute pancreatitis [7].

Although the pathogenesis of black esophagus remains unknown, most investigators have suggested an ischemic origin based on histopathologic and clinical data. This pathology usually develops in elderly patients with vascular disorders that render them vulnerable to ischemic injury [4]. The association of esophageal injury with a low flow state and the rapid resolution of the esophageal lesion after hemodynamic stabilization suggest that temporary reduction in esophageal blood perfusion can result in extensive esophageal necrosis [7]. Another theory evokes transient gastric outlet obstruction with accumulation of large volumes of liquid in the stomach with prolonged esophageal reflux [9]. The differential diagnosis includes melanosis, malignant melanoma, pseudomelanosis, acanthosis nigrans, adverse drug effects (quinidine and tetracycline), and infection. Complications include perforation, stricture, microbial superinfection and death [7].

To date, there are no known treatments for black esophagus. The current recommendation is to intensively treat the patient's comorbidities, optimize vascular perfusion, aggressively suppress acid production, and treat esophageal infections, if present [8]. Surgical intervention in patients with AEN is reserved for perforated esophagus with resultant mediastinitis and abscess formation. The overall mortality in the largest case review to date was 32%. The high mortality was most frequently secondary to the seriousness of comorbid disease states [7]. In our case, patient presented no further hematemesis or melena, the mortality was due to severity of sepsis despite resuscitative measures.

## Conclusion

Acute necrotizing esophagitis is a serious clinical condition. It should be suspected in those with upper gastro intestinal bleed and particularly the elderly with comorbid illness. The pathogenesis remains unknown; an ischemic origin appears likely in many patients. Its mortality remains high. Early diagnosis with endoscopy and active management will lead towards an improvement in patient outcome.
